# A strategy to improve skills in pharmaceutical supply management in East Africa: the regional technical resource collaboration for pharmaceutical management

**DOI:** 10.1186/1478-4491-6-30

**Published:** 2008-12-23

**Authors:** Lloyd Matowe, Paul Waako, Richard Odoi Adome, Isaac Kibwage, Omary Minzi, Emile Bienvenu

**Affiliations:** 1 RPM Plus Program, Center for Pharmaceutical Management, Management Sciences for Health, 4301 N. Fairfax Drive, Arlington, VA 22203, USA; 2 Department of Pharmacology and Therapeutics, Makerere University, Kampala, Uganda; 3 Department of Pharmacy, Makerere University, Kampala, Uganda; 4 School of Pharmacy, University of Nairobi, Nairobi, Kenya; 5 School of Pharmacy, Muhimbili University College of Health and Allied Sciences, Dar es Salaam, Tanzania; 6 Department of Pharmacy, Faculty of Medicine, National University of Rwanda, Butare, Rwanda

## Abstract

**Background:**

International initiatives such as the Global Fund to Fight AIDS, Tuberculosis and Malaria, the President's Emergency Plan for AIDS Relief and the President's Malaria Initiative have significantly increased availability and access to medicines in some parts of the developing world. Despite this, however, skills remain limited on quantifying needs for medications and ordering, receiving and storing medications appropriately; recording medications inventories accurately; distributing medications for use appropriately; and advising patients on how to use medications appropriately. The Regional Technical Resource Collaboration for Pharmaceutical Management (RTRC) has been established to help address the problem of skills shortage in pharmaceutical management in East Africa.

**Methods:**

The initiative brings together academic institutions from four East African countries to participate in skills-building activities in pharmaceutical supply management. The initiative targeted the institutions' ability to conduct assessments of pharmaceutical supply management systems and to develop and implement effective skills-building programmes for pharmaceutical supply chain management.

**Results:**

Over a two-year period, the RTRC succeeded in conducting assessments of pharmaceutical supply management systems and practices in Kenya, Rwanda, Tanzania and Uganda. In 2006, the RTRC participated in a materials-development workshop in Kampala, Uganda, and contributed to the development of comprehensive HIV/AIDS pharmaceutical management training materials; these materials are now widely available in all four countries. In Tanzania and Uganda the RTRC has been involved with the training of health care workers in HIV/AIDS pharmaceutical management. In Kenya, Tanzania and Uganda the RTRC has been conducting operations research to find solutions to their countries' skills-shortage problems. Some of the interventions tested include applying and evaluating the effectiveness of a novel skills-building approach for pharmaceutical supply management.

**Conclusion:**

Nurturing collaboration between regional institutions in resource-limited countries to build in-country skills in pharmaceutical supply management appears to be an effective intervention. Support from local programmes and technical assistance from organizations and institutions with the necessary expertise is critical for success, particularly at inception. The skills acquired by local institutions can be incorporated into both pre-service and in-service teaching curricula. This ensures long-term availability of skills in-country. The ability of trained institutions to mobilize their own resources for skills-building activities is crucial for the success and sustainability of these programmes.

## Background

International initiatives such as the Global Fund to Fight AIDS, Tuberculosis and Malaria, the President's Emergency Plan for AIDS Relief and the President's Malaria Initiative have significantly increased availability and access to medicines in some parts of the developing world. However, these increases in the supply of medications are straining systems that are already weak in pharmaceutical supply management. Weaknesses include inadequate capacity and skills to quantify needs for medications or to order, receive and store medications appropriately and to record medications inventories accurately.

In addition, increased supply of medicines often means increased opportunity for inappropriate use [1-3]. Inappropriate patterns of drug use behaviour can result in unsafe pharmaceutical use, waste of resources, non-compliance and excessive adverse drug reactions [3].

Training has been documented as the main intervention to improve pharmaceutical management skills in developing countries [4]. However, there is abundant evidence that training alone is often insufficiently effective to change practice [5-7]. And where traditional training methods produce positive results, the change has been reported as transient and unsustainable [5,8]. Other interventions are often necessary to reinforce training as a behaviour change strategy [8]. Locally-based interventions that include many stakeholders have been reported as effective in producing sustainable change [8,9]. In this paper we describe how, with a focus on sustainability, acceptability and achieving long-term capacity, Management Sciences for Health's RPM Plus Program supported Makerere University in Uganda to develop and foster a regional network of academic institutions in East Africa to build in-country and regional capacity for pharmaceutical supply management.

## Methods

### RTRC: What is it?

**Figure 1:**
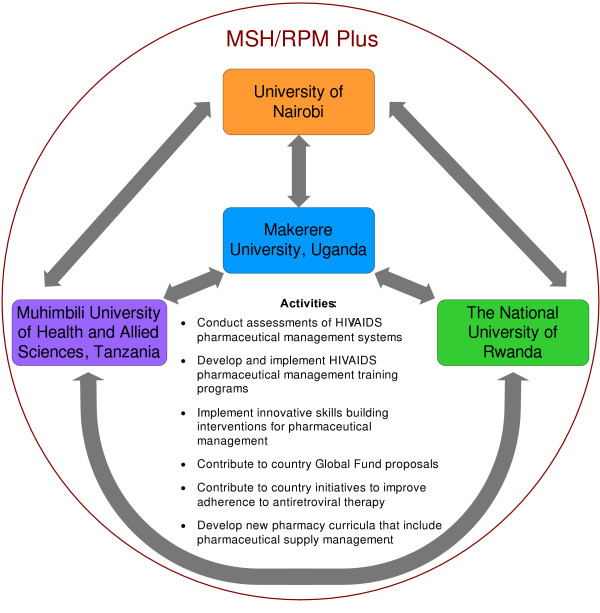
**The Regional Technical Resource Collaboration for Pharmaceutical Management concept**.

The RTRC is a network of academic and other institutions brought together to build in-country capacity in pharmaceutical supply management in four East African countries. The initiative includes Makerere University in Uganda, the University of Nairobi in Kenya, the National University of Rwanda and Muhimbili University of Health and Allied Sciences in Tanzania. The concept, represented diagrammatically in Figure 1, is modelled on lessons learnt from the International Network for Rational Use of Drugs [9-11]. The RTRC is a cooperative organization whose ultimate goal is to build the skills of health care workers in pharmaceutical supply management.

The RTRC consists of core groups in each of the four countries. Each country core group is multidisciplinary and draws participants from other in-country institutions apart from academic institutions. In addition to academicians, core group members include pharmacists, social scientists, policy-makers, and programme implementers with responsibility for pharmaceutical supply management. Each country core group consists of 8 to12 people and is coordinated at the academic institutions named above.

In Uganda, the RTRC is coordinated by Makerere University's Departments of Pharmacology & Therapeutics and the Department of Pharmacy. The Uganda RTRC works with and complements existing country initiatives such as the Academic Alliance, which runs programmes for HIV/AIDS treatment and care. The Kenya RTRC is based at the School of Pharmacy at Nairobi University. Other institutions involved in the initiative in Kenya include the Kenya Medical Research Institute, the Ministry of Health (MOH) and the National AIDS and STDs Control Program (NASCOP). In Tanzania, the RTRC is based in the School of Pharmacy at Muhimbili University of Health and Allied Sciences. Other participating institutions in Tanzania include the MOH, the National AIDS Control Program (NACP) and the Tanzania Food and Drug Administration (TFDA). In Rwanda, the RTRC is based at the School of Public Health and the Department of Pharmacy in the School of Medicine at the National University of Rwanda. Other participating departments in Rwanda include the MOH and the Treatment and AIDS Research Centre (TRAC).

### Regional coordination and technical assistance

Regionally, the RTRC is coordinated by Makerere University. Makerere University's central role includes coordinating regional activities, identifying and mobilizing resources for regional activities and centralized monitoring and evaluation. Technical assistance for the RTRC is provided by Management Sciences for Health's RPM Plus Program. RPM Plus is a United States Agency for International Development-supported programme that has vast international experience on addressing pharmaceutical supply management challenges. RPM Plus' areas of expertise include designing and applying tools to understand pharmaceutical management systems, providing technical guidance in strategy development, programme implementation, training local health care staff to improve the efficiency of pharmaceutical supply systems and working with policy-makers, researchers and managers in the public and private sectors to implement new and proven interventions. Using limited resources, RPM Plus worked closely with the RTRC to build the latter's skills and competences in many areas of pharmaceutical supply management.

### Why was it formed?

The RTRC was formed to build regional and in-country capacity in pharmaceutical management, including addressing bottlenecks in the commodities supply chain. Capacity-building activities are linked with national needs. Activities are identified, designed and conducted according to their relevance to national priorities and whether they can be linked to country-level interventions. For example, the development of training programmes for HIV/AIDS pharmaceutical management in Uganda (to be discussed later) was linked to the NACP's goal to build the skills of facility-level health care workers involved in the supply management of HIV/AIDS medications and related commodities.

## Results

### Assessment of HIV/AIDS pharmaceutical supply management systems

One of the main activities undertaken by the RTRC was conducting assessments of the HIV/AIDS pharmaceutical supply management systems in the four countries. The assessments sought to determine the capacity of the health care systems of the four countries to select, quantify, distribute and appropriately use ARVs and related commodities; determine the categories of health care workers involved in the supply chain management of HIV/AIDS pharmaceuticals; and assess their knowledge, skills and practices.

The results of the assessments showed that problems with ART commodities-supply management existed widely in Kenya, Rwanda, Tanzania and Uganda. These problems ranged from the inability of the existing systems to adequately handle scale-up programmes to lack of readiness of the workforce to efficiently use and manage large supplies of antiretrovirals, including inadequate capacity to quantify needs and distribute the medications and inappropriate medication-distribution practices. Inadequate skills were cited as the main reason for the identified problems in all four countries. There was thus a need to build skills in HIV/AIDS pharmaceutical supply management in all four countries. Skills-building processes that included local institutions were preferred, as these would cover wider geographical areas. These were also regarded as more sustainable. The methodology and comprehensive results of this assessment have been described elsewhere [12].

### Developing HIV/AIDS pharmaceutical management training materials

In 2006, the RTRC participated in a materials-development workshop in Kampala, Uganda, and contributed to the development of comprehensive HIV/AIDS pharmaceutical management training materials. The workshop, facilitated by RPM Plus, resulted in the development of generic HIV/AIDS pharmaceutical management training materials. These materials can be easily adapted for local use to support ART programmes. Following the development of the materials, Kenya, Tanzania and Uganda succeeded in adapting them for local use. These materials have been widely used for HIV/AIDS pharmaceutical management training in all four countries. Other countries, including Ghana, Liberia and Namibia, have since adapted these materials for local use.

### Training on HIV/AIDS pharmaceutical management

In Uganda and Tanzania the RTRC has been actively participating in the training of health care workers in HIV/AIDS pharmaceutical management. In Uganda, this training has been supported by the NACP, the World Health Organization (WHO), Catholic Relief Services and other intergovernmental or nongovernmental organizations. In Tanzania, the training has been supported by the NACP, WHO, the National Medical Stores and NGOs. To date, the Uganda RTRC has conducted three national training courses on HIV/AIDS pharmaceutical management. This translates to more than 100 health care workers involved in managing commodities at facilities providing ART services. In Tanzania, the RTRC has trained more than 60 health care workers from different parts of the country on HIV/AIDS pharmaceutical management. In all four countries, many organizations, including the NACP, WHO, MSD and others involved in the management of ART commodities, have routinely used the RTRC as consultants or as facilitators for courses on pharmaceutical supply management.

### Conducting operations research

The RTRC has been involved in conducting operations research to find solutions for their countries' skills-shortage problems. Some of the interventions tested include applying and evaluating the effectiveness of the Monitoring-Training-Planning (MTP) approach as a skills-building approach for pharmaceutical supply management. MTP is an innovative approach to capacity building that empowers participants to solve their own problems [13,14]. It is a simple, low-cost intervention that seeks to build the skills of participants at their workplaces.

The RTRC applied MTP to 34 facilities providing ART services in Kenya, Tanzania and Uganda. The process involved working with NACP to prioritize and select facilities for skills-building in each of the three countries. Workers from the selected facilities were invited to attend a skills-building workshop at a central place, where results of a prior assessment of ART pharmaceutical management practices at their sites were discussed and solutions suggested. Each facility then worked on implementing suggested solutions, developing time lines for implementation and setting targets for improvement. The RTRC, together with NACP, conducted follow-up visits to each of the facilities every six weeks for a total of three visits. The results showed that MTP is an effective and sustainable intervention to build the skills of low-level health care workers managing commodities at ART facilities. Plans are currently under way to scale up MTP in all three countries.

## Discussion

The RTRC initiative demonstrated a capacity-building model that is effective and has tremendous potential to be sustainable. Potential for sustainability is enhanced by the fact that participating institutions and groups are supported through funding for commissioned activities and products, rather than grants to support non-specific capacity building. For example, funding for Makerere University to conduct HIV/AIDS pharmaceutical management training was received from NACP, WHO and the Catholic Relief Services. Focusing on a service-oriented approach and using local institutions to address country-specific needs helps to ensure long-term availability of skills. From January 2006 to December 2007 the RTRC mobilized more than USD 400 000 to support in-country programmes. Table 1 shows the resources mobilized by the initiative over a period of two years.

**Table 1:** Resources generated by the RTRC between January 2006 and December 2007

**Activity**	**Countries**	**Source of funding**	**Amount (USD)**
Assessment of ART commodity-management practices in Uganda, Kenya, Tanzania and Rwanda	Uganda, Kenya, Tanzania, Rwanda	USAID/RPM Plus Program	100 000
National HIV/AIDS pharmaceutical supply management training programmes	Uganda, Tanzania	National AIDS-control programmes, WHO, Catholic Relief Services, Medical Stores Department, Rakai Health Sciences Program Children AIDS Fund	80 000
HIV/AIDS pharmaceutical supply management training consultancies	Uganda, Kenya, Tanzania	Various in-country organizations, e.g. MSD in Tanzania, NASCOP in Kenya	60 000
Evaluating MTP as a skills-building approach for HIV/AIDS pharmaceutical management	Uganda, Kenya, Tanzania	USAID/RPM Plus Program	90 000
Conducting locally-based Drugs and Therapeutics Committee Course in Uganda and Tanzania	Uganda, Tanzania	Fee-paying courses	80 000

**Total**			**410 000**

A number of junior members of the academic staff within the aforementioned institutions were targeted for capacity building. This allowed the system to build a significant pool of professionals with skills and competences in pharmaceutical supply management. At Tanzania's Muhimbili University College of Health and Allied Health, 10 members of the academic staff have developed competences and skills in pharmaceutical management. These include three senior staff members and seven junior staff members. Makerere University in Uganda has 13 staff members who have developed competences in pharmaceutical management, including five senior staff members and nine junior staff members. Makerere University has gone further and has hired three junior members of academic staff from the proceeds of pharmaceutical management activities. The ability to build the skills of staff members and to hire new staff demonstrates the long-term potential of the model.

Following the development of the training materials and the training of a number of their academic staff members in pharmaceutical supply management, Makerere University's Department of Pharmacy has now adapted various components into its pre-service pharmacy curriculum. In addition, the schools of pharmacy in both Tanzania and Uganda have plans to develop Master's of Science programmes in pharmaceutical supply management that draw largely from the initiative. In Rwanda, the Department of Pharmacy at the National University of Rwanda has revised their pre-service curriculum to include components of pharmaceutical supply management. The development of a new curriculum and the establishment of new courses in pharmaceutical supply management also demonstrate the potential of this approach to be sustainable.

### Challenges

The main challenge faced by the programme was a severely understaffed academic system. Involving academic staff members in service-delivery activities who were already overburdened with teaching commitments was always going to present a challenge. This barrier was ameliorated by the inclusion of other institutions in the scheme and the ability of the institutions to build the skills of junior staff members.

## Conclusion

Nurturing collaboration between regional institutions in resource-limited countries to build in-country skills in pharmaceutical supply management appears to be an effective intervention. Support from local programmes and technical assistance from organizations and institutions with the necessary expertise is critical for success, particularly at inception. The skills acquired by local institutions can be incorporated into both pre-service and in-service teaching curricula. This ensures long-term availability of skills in-country. The ability of trained institutions to mobilize their own resources for skills-building activities is crucial for the success and sustainability of the programme.

## Competing interests

The authors declare that they have no competing interests.

## Authors' contributions

LM coordinated the RTRC for Management Sciences for Health, provided technical assistance to the initiative and coordinated and helped to draft the manuscript. PW and RO coordinate the RTRC at Makerere University and helped to draft the manuscript. IK, OM and EB coordinate the RTRC in Kenya, Tanzania, and Rwanda, respectively, and all contributed to the manuscript.
